# Ultra-Precision Machining of a Compound Sinusoidal Grid Surface Based on Slow Tool Servo

**DOI:** 10.3390/ma11061001

**Published:** 2018-06-13

**Authors:** Shijun Ji, Jianfeng Li, Ji Zhao, Mei Feng, Changrui Sun, Handa Dai

**Affiliations:** School of Mechanical Science and Engineering, Jilin University, Changchun 130025, China; jishijun97@126.com (S.J.); ljf16@mails.jlu.edu.cn (J.L.); jzhao@jlu.edu.cn (J.Z.); suncr14@mails.jlu.edu.cn (C.S.); dhd@jlu.edu.cn (H.D.)

**Keywords:** compound sinusoidal grid surface, slow tool servo, ultra-precision machining, tool nose radius compensation

## Abstract

Compound sinusoidal grid surface with nanometric finish plays a significant role in modern systems and precision calibrator, which can make the systems smaller, the system structure more simple, reduce the cost, and promote the performance of the systems, but it is difficult to design and fabricate by traditional methods. In this paper, a compound freeform surface constructed by a paraboloidal base surface and sinusoidal grid feature surface is designed and machined by slow tool servo (STS) assisted with single point diamond turning (SPDT). A novel combination of the constant angle and constant arc-length method is presented to optimize the cutting tool path. The machining error prediction model is analyzed for fabricating the compound sinusoidal grid surface. A compound sinusoidal grid surface with 0.03 mm amplitude and period of 4 is designed and cutting process is simulated by use of MATLAB software, machining experiment is done on ultra-precision machine tool, the surface profile and topography are measured by Taylor Hobson and Keyence VR-3200, respectively. After dealing with the measurement data of compound freeform surface, form accuracy 4.25 μm in Peak Village value (PV), and surface roughness 89 nm in Ra are obtained for the machined surface. From the theoretical analysis and experimental results, it can be seen that the proposed method is a reasonable choice for fabricating the compound sinusoidal grid surface.

## 1. Introduction

When compared with traditional elements, compound freeform surface elements can obviously make the systems smaller, make the system structure much simpler, reduce the cost, and promote the performance of the systems, and it has drawn more attention in recent years [1,2]. The compound sinusoidal grid surface is a typical shape of freeform surface with a wide range of application in many fields, such as new energy, aerospace, illumination, and biomedical engineering et al. [3], due to the specificity of the application fields, it is required to be with sub-micrometer form accuracy PV and nanometer surface roughness Ra finish [4,5].

Nowadays, many researches have concentrated on how to design and manufacture a single simple piece of optical surface, such as spherical or aspherical surface, off-axis surface, and simple micro-structure surface. Ying Cheng et al. manufactured an off-axis optical reflective integrator by ultra-precision machining, the advancement in this study is to reduce the stepwise discontinuity among the facets to make it applicable for turning, and the off-axis optical reflective integrator is more simple in structure and smaller in size when compared with existing refractive integrators, and simulations and experiments show that above 90% of illumination uniformity can be achieved using this new design [6]. Xiaodong Zhang et al. proposed a coordinate transformation machining method aided with STS which make the machining feasible for off-axis aspheric mirrors. Reducing the ratio of sag height to diameter, the cutting path is designed by the coordinate transform. They not only machined the off-axis aspheric mirrors, but also fabricated freeform prisms to prove that the proposed method works well for complex surfaces [7]. Two different ultra-precision manufacturing methods for off-axis parabolic mirror with single point diamond turning are analyzed and compared by Shijun Ji et al., the two methods are that turning the cylindrical blank by revolving around the axis of the parabolic surface and revolving around the axis of cylindrical surface, besides, the kinematic analyses of two linear axes (X, Z) are studied, the tool path generation and installation errors are analyzed under two different conditions. Experimental results of off-axis parabolic mirror show that the surface machined with turning the cylindrical blank by revolving around the axis of parabolic surface has better machining quality than the other one [8]. The cylindrical coordinate micromachining method for ultra-precision cutting of sinusoidal surfaces was studied by Xiaodong Zhang et al., a three-axis turning machine is used and a spiral curve NC path is adopted, meanwhile, they proposed a method to optimize the tool geometry and to simulate the cutting process, a sinusoidal surface with 5.54 nm in Ra are achieved in their study [9]. Hong Lu et al. fabricated an sinusoidal grid surface by diamond machining assisted with fast tool servo (FTS) system, for the machining of sinusoidal grid surface, they designed the compact mechanism structure and tested the dynamic performance of the FTS, and the machining results show that the dimension of sinusoidal grid change with the variation of the FTS machining condition, which indicates that this method is suitable to machine various sorts of sinusoidal feature surface [10]. Xiaoqin Zhou et al. investigated a double-frequency elliptical vibration cutting apparatus that combines FTS and elliptical vibration cutting for freeform surface diamond machining, the apparatus is designed to be a flexure hing structure and driven by two piezoelectric actuators to generate tool motions, it does well in machining hard materials. A 40Cr die steel workpiece is manufactured, and the experimental results show better surface quality than conventional fabrication methods [11]. Lingbao Kong et al. investigated the generated surface modeling and characterization with respect to FTS fabrication, who established a model to predict the surface generation based on a mechanism of FTS and other factors, meanwhile, a surface matching based method was proposed to characterize the microlens arrays surface quality, which measured the microlens arrays as a whole instead of a single lens evaluation. The experiments results agree well with the prediction results [12]. Precision machining of ‘water-drop’ surface was proposed by Quanli Zhang et al. using single point diamond grinding, that investigated the linkage movement of the machine to grind the ‘water-drop’ surface and presented methods to compensate the alleviation of the center dent error, based on the proposed methods, the form accuracy and surface finish of ‘water-drop’ surface reaches 0.46 μm (PV), 6 nm (S_a_) on binderless tungsten carbide [13]. Shijun Ji et al. fabricated a large amplitude sinusoidal ring surface that was based on STS, the kinematic characteristic of machine axes is analyzed to assess the feasibility of the fabrication process. The tool path generation, tool geometry optimization, and tool radius compensation are also investigated for the fabrication of the sinusoidal ring surface, an experimental sample with 0.4 mm amplitude sinusoidal ring surface is successfully machined, and a form accuracy 0.247 μm in PV and surface roughness 7.5 nm in Ra is obtained [14]. Szymon Wojciechowski et al. proposed a method to minimize the cutting forces and vibration during ball end milling. They found the inclination angle of surface and tool’s overhang length were closely relate to the cutting forces and vibrations. The experimental results demonstrate that the optimized values of surface inclination angle and tool’s overhang length can greatly improve the surface quality [15]. Jayant Kumar et al. studied the thermal effects in single point diamond turning by proposing a mathematical model to evaluate the net residual heat, which was transferred in workpiece. A cylindrical workpiece is demonstrated with respect to the rate of heat transfer and some other aspects, the simulation model and sets of experiments based on the mathematical model were conducted, and the experiment results surface accuracy were significantly improved [16]. S. Wojciechowski et al. presented a method to reduce the milling forces and to improve the machining efficiency, they measured the cutting forces and process efficiency according to the cutting parameters and the surface inclination angle, rely on the multi-criteria optimization of cutting forces and efficiency, and the minimisation of a total utility function, the experiment results show that the cutting conditions had a great effect on cutting forces, and they obtained the optimal parameters of cutting speed and surface inclination angle for machining [17]. From the above analyses, it can be seen that there are many studies focused on how to design and fabricate a single simple piece of optical surface. While, few studies have concentrated on compound freeform surface. Xiaodong Zhang et al. machined compound eye lens by STS, on one hand, they investigated the mathematic and optical analysis of compound eye lens, which is based on proposing a grid machining method for the fabrication of a compound eye lens. On the other hand, in order to guarantee the surface accuracy, the off-centering machining configuration is presented to avoid the shape distortion. Based on the proposed methods, the compound eye lens is machined on a plane, and a compound freeform surface that is constructed by sphere base surface and compound eye lens was also successfully fabricated, while they did not show the surface accuracy and just indicated that the method presented was suitable for the compound eye lens with curve substrate [18].

By analyzing the previous research work, it can been seen that most of research work concentrated on manufacturing single freeform surfaces, few researches investigated on machining compound freeform surfaces. In this paper, a compound freeform surface is designed by combining a paraboloidal base surface and a sinusoidal grid feature surface, optimization of cutting tool path, tool nose radius compensation, and error analysis are done in Section 2. Experiments and discussion is given in Section 3. It is concluded in Section 4. 

## 2. Basic Theory and Methodology of Compound Freeform Surface Model

### 2.1 Compound Freeform Surface Model Establishing

**Figure 1:**
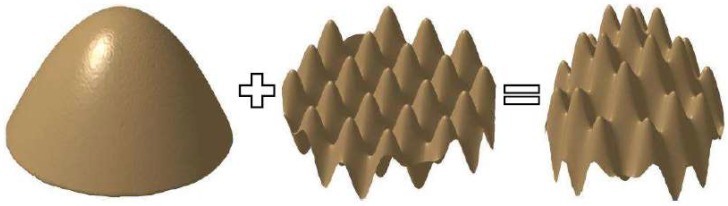
Illustration of compound freeform surface combination theory.

Compound freeform surface is different from traditional freeform surface in generating, traditional surface is confirmed by an equation or a series of equations, compound freeform surface is obtained by combining double or more freeform surfaces, and Figure 1 shows the compound freeform surface combination theory.

From the above illustration of freeform surface, we can reach a conclusion that compound freeform surfaces compose of a base surface and other one feature surface or more feature surfaces, which compares with the traditional single freeform surface. In view of the complex composition of compound freeform surface, the tool path will be more complicated and more challenges will be faced. In this paper, authors select the compound sinusoidal grid surface to be fabricated, which consists of a paraboloidal base surface and a sinusoidal grid feature surface.

#### 2.1.1. Base Surface Model Establishing 

**Figure 2:**
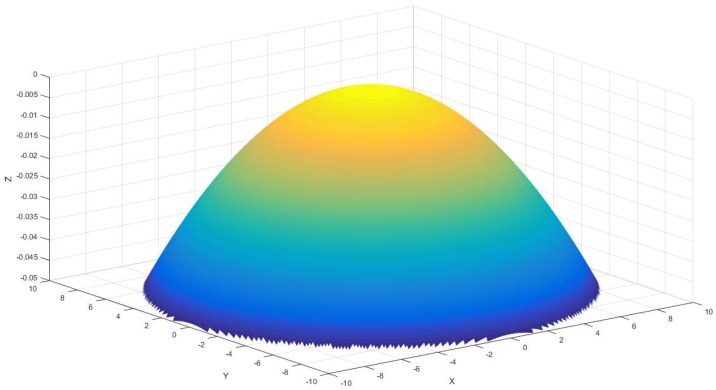
Base surface of the compound sinusoidal grid surface.

Compound sinusoidal grid surface include two features, paraboloidal surface and sinusoidal grid surface. In this section, a base surface paraboloidal surface model will be established, the equation of which is described, as follows:

(1) $$Z_{1}=\sqrt{r_{1}{}^{2}-x^{2}}+\sqrt{r_{2}{}^{2}-y^{2}}$$

where $r_{1}$ and $r_{2}$ are used to confirm the curvature in X direction and Y direction, respectively. The model of Equation (1) is shown in Figure 2, and it is a paraboloidal surface as the base surface of compound sinusoidal grid surface. 

This section mainly describes the process of paraboloidal surface establishing, which is the first step to build compound sinusoidal grid surface.

#### 2.1.2. Feature Surface Model Establishing

**Figure 3:**
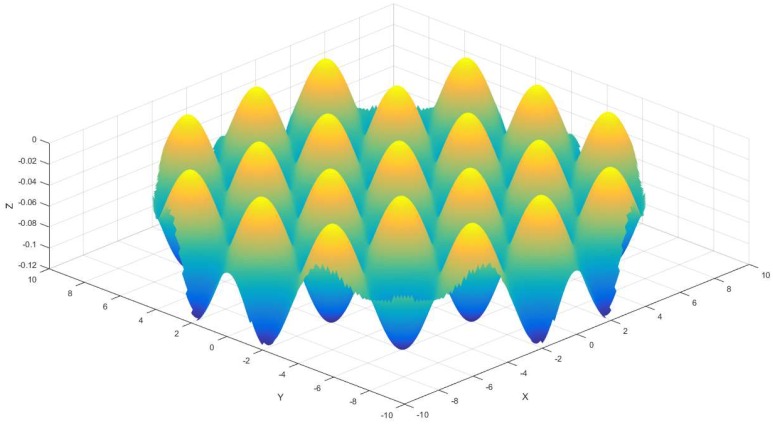
Sinusoidal grid surface.

From the previous sections, we can know that a compound freeform surface is composed of two or more features; this section will present the second feature surface that is named sinusoidal grid surface, which can be expressed as Equation (2).

(2) $$Z_{2}=F_{1}+F_{2}$$

where $F_{1}$ and $F_{2}$ can be written as: $F_{1}=h_{1}\cdot \sin(\omega_{1}\cdot x)$, $F_{2}=h_{2}\cdot \sin(\omega_{2}\cdot y)$. $h_{1}、h_{2}$ is the amplitude of the sinusoidal grid surface along X direction and Y direction, respectively, $\omega_{1}、\omega_{2}$ is used to determine the period of the sinusoidal grid surface along X direction and Y direction, respectively, and $F_{1}$, $F_{2}$ is orthogonal mutual. The simulation consequence for sinusoidal grid surface is shown in Figure 3.

#### 2.1.3. Compound Sinusoidal Grid Surface Establishing

Base surface and feature surface have been established up to now, and the following research will concentrate on integral compound freeform surface generation. According to Figure 1, a compound freeform surface is made up of two or more sections, this paper desires to fabricate a compound freeform surface that consists of a paraboloidal base surface and a sinusoidal grid feature surface. For the sake of obtaining the ultimate compound surface, the essential procedure is to recombine the double surface, this step can be obtained, as follows:

(3) $$Z=Z_{1}+Z_{2}$$

**Figure 4:**
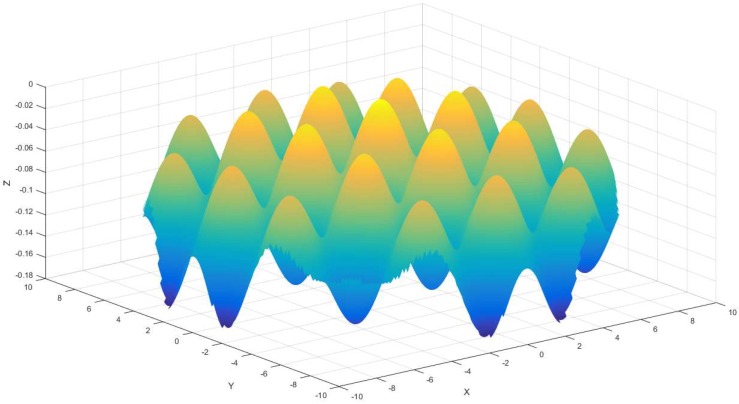
Compound sinusoidal grid surface.

According to Equation (3), the calculated compound sinusoidal grid surface consequence is shown in Figure 4.

As displayed in Figure 4, the compound sinusoidal grid surface is obtained by means of recombining base surface and feature surface, which are built in advance. The previous works are the bases of tool path generation that will be realized in the coming section. 

### 2.2. Tool Path Generation

**Figure 5:**
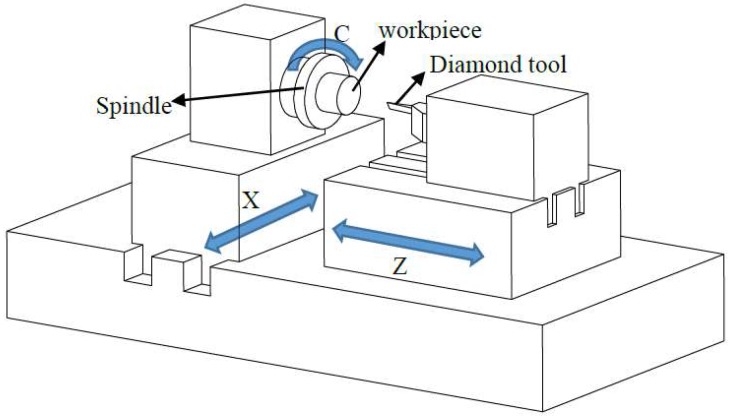
Configuration of the machining system.

Figure 5 shows a typical three-axis ultra-precision turning machine tool. There are two straight line motions, X-axis, Z-axis, and one rotation motion, C-axis, in the machine system, which is controlled by slow tool servo (STS) technology. In the slow tool servo machine, the work-piece or the fixture is mounted on the spindle. When the spindle turns a fixed angle, the tool will move to a determined position, according to the special position relationship between the tool and work piece. This motion mechanism has the competency of fabricating the freeform surfaces with a complex shape.

#### 2.2.1. Constant Angle Cutting Path

**Figure 6:**
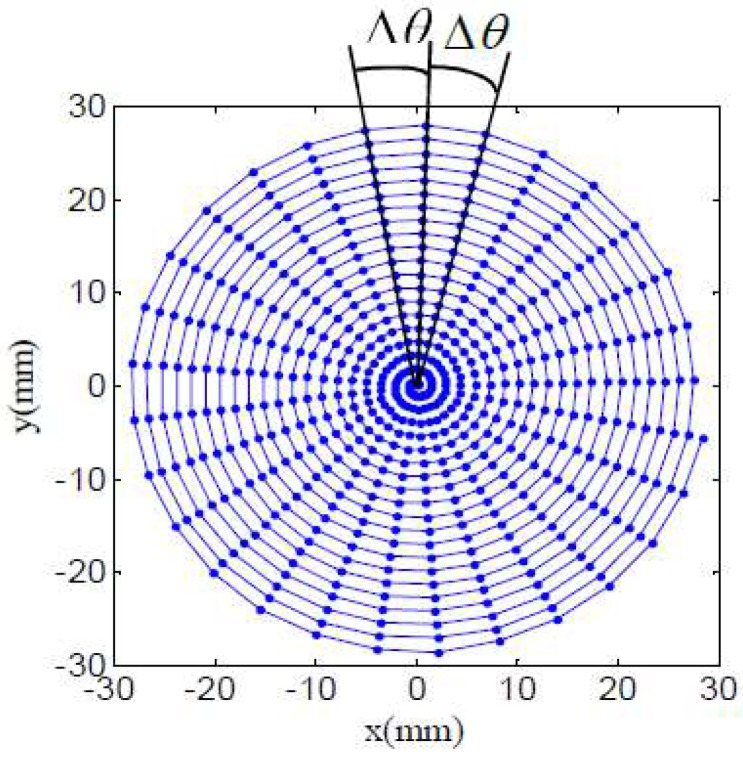
Constant angle cutting path.

In this paper, a compound freeform surface on a cylindrical end face was manufactured, there are two cutting path generation methods including constant angle and constant arc-length methods. As is depicted in Figure 6, it is constant angle Δθ tool path, the outstanding characteristic is that every central angle is a constant value between adjacent double points, and the points on the cutting path are all in a plane, which are named data cloud as the base of tool contact points generation. 

For the sake of achieving high accuracy of the compound freeform surface, it is critical to choose an appropriate cutting path. As for the constant angle tool path that is displayed in Figure 3, the main characteristic is that every central angle is a constant value between an adjacent double points, however, the arc-length between an adjacent point is not, the outer area arc-length is longer than that at the central region, thus the central area surface accuracy is better than the outer region. In order to get high accuracy compound freeform surface finish, the outer area of it is the key factor, and a smaller angle Δθ must be chosen to control the compound freeform surface accurately. On the one hand, the tool path points at the central region will be too dense, it will cost too much time and will have low efficiency. On the other hand, a smaller angle of cutting path demands a high spindle rotation resolution. On the contrary, due to the variable Δθ is a constant value, so that it requires a lower dynamic performance of spindle rotation. 

#### 2.2.2. Constant Arc-Length Cutting Path

**Figure 7:**
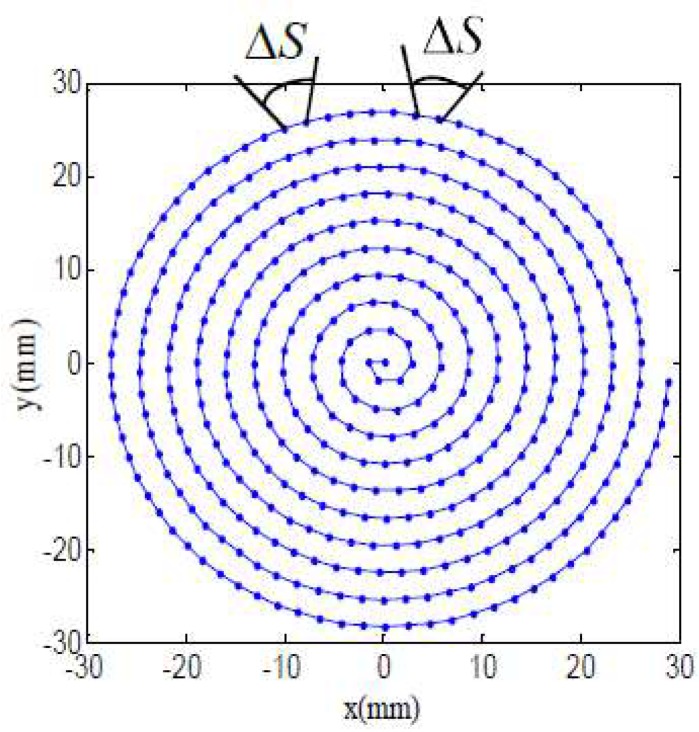
Constant arc-length cutting path.

When compared with constant angle tool path, the main characteristic of constant arc-length Δ*S* cutting path is that each arc between double adjacent cutting points is a constant value, as shown in Figure 7, and the points on the cutting path are all in a plane, which are named data cloud as the base of tool contact points generation. The defects of this method mainly include the following aspects, the arc-length between every two points in the central region takes up a bigger angle value than the outer arc-length, and thus the outer area surface accuracy is superior to the central region. Besides, the spindle rotation speed is various as fabricating the central and outer area, and there would be an acceleration at spindle speed. 

**Figure 8:**
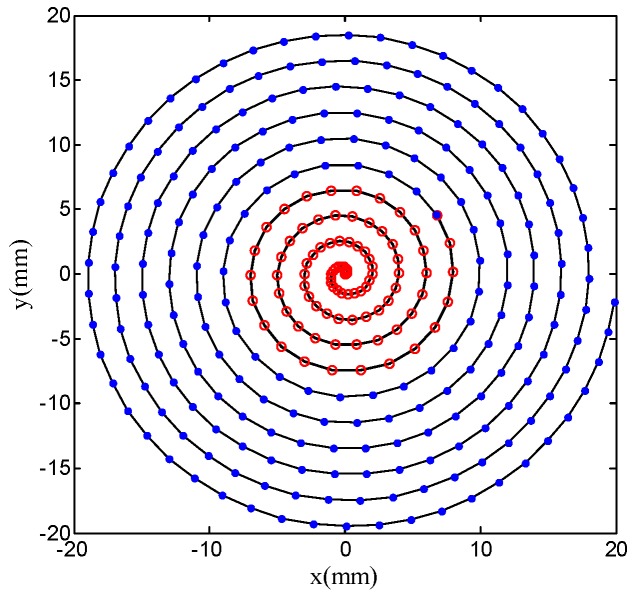
The recombination result of constant angle and constant arc-length method.

Depending on the above analysis, there is a high accuracy at the central region with constant angle cutting path, and the surface accuracy will be superior in outer area with constant arc-length cutting path, so this paper proposed a method that combines the constant angle and the constant arc-length means that the central region of compound sinusoidal grid surface adopts constant angle cutting path and the outer area employs a constant arc-length cutting path. The recombination result of constant angle and constant arc-length method is displayed as Figure 8. 

**Figure 9:**
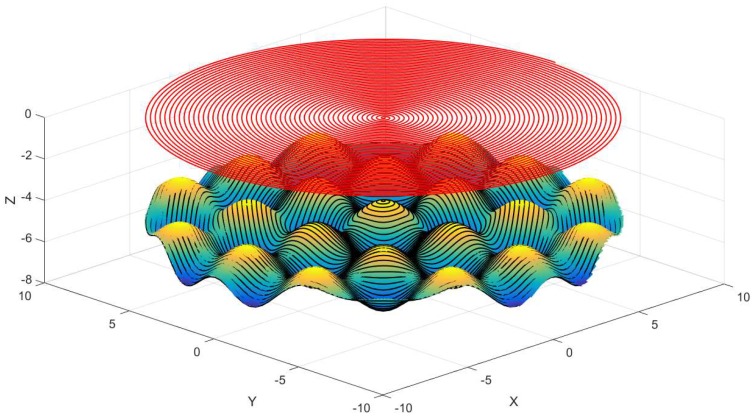
Illustration of tool contact points generation.

As is shown in Figure 8, the central region spiral path in red is the constant angle cutting path and the outer area spiral path in blue is constant arc-length cutting path. All points in Figure 8 is named data cloud used to generate tool contact points, which are built in advance by means of projecting the data cloud on the compound sinusoidal grid surface, thus the accuracy of cutting path will impact the compound freeform surface quality directly. Tool contact points generation is illustrated, as in Figure 9.

### 2.3. Tool Parameters Optimum

**Figure 10:**
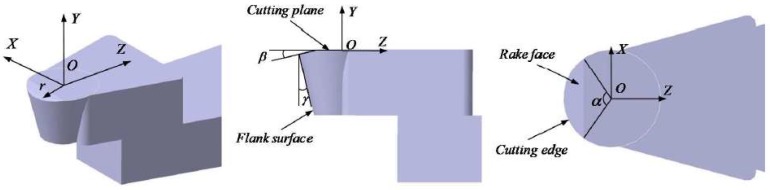
Illustration of tool geometry.

The previous works have determined the compound sinusoidal grid surface, cutting path, and tool contact points. Ultra-precision machining also requires a suitable diamond tool with optimal parameters including tool nose radius *r*, clearance angle γ, and wrap angle *α*, which is shown as Figure 10.

Tool geometry parameters have a great influence on the machined surface feature, thus a series of fit tool geometry parameters is essential to guarantee the high accuracy of compound freeform surface finish. The subsequent search is about to concentrate on tool geometry analysis.

**Figure 11:**
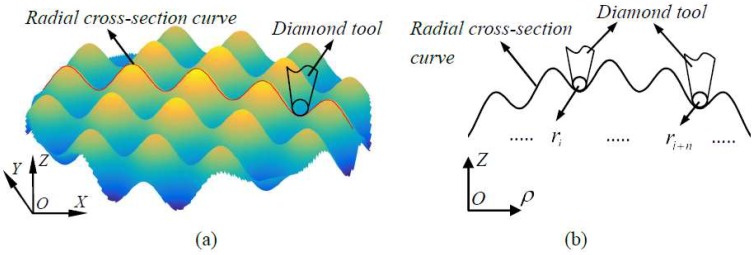
Illustration of geometry between tool nose radius and radial cross-section curve. (a) The surface radial curve for tool nose radius optimum; (**b**) Geometry relation between tool nose radius and surface radial curve curvature radius.

The first study is about tool nose radius optimum, because the desired compound sinusoidal grid surface is concave-convex severely, and the corresponding cutting path is concave-convex. It will do harm to the compound freeform surface finish accuracy with an unfit tool nose radius. Figure 11 illustrates the geometry between tool nose radius and surface radial curve, Figure 11**a** shows the way that obtains the surface radial curve for tool nose radius optimum, Figure 11b demonstrates the geometry relation between tool nose radius and surface radial curve curvature radius, it is feasible under the condition of the tool nose radius is smaller than the minimum value of radial curve among each cutting point. 

**Figure 12:**
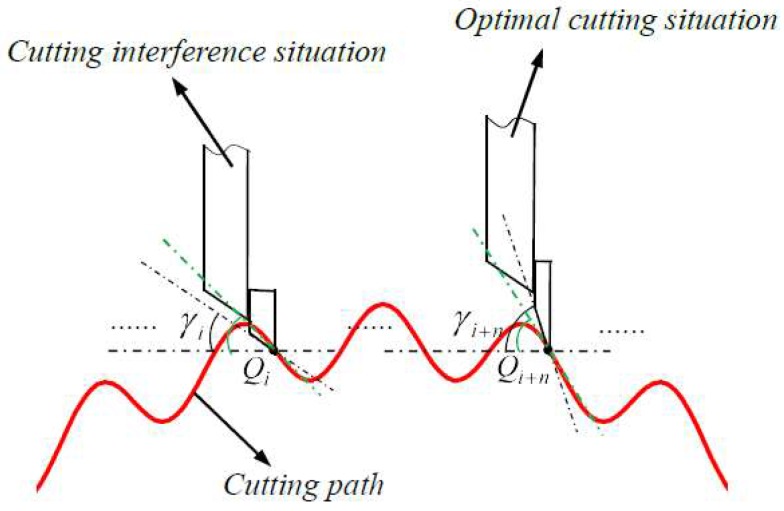
Demonstration of clearance angle optimum.

Clearance angle is another indispensable factor for optimum of tool geometry, it has a considerable impact on machined surface, as shown in Figure 12. As for the left case, $\gamma_{i}$ is tool clearance angle, and inclination angle for the corresponding cutting point $Q_{i}$ is less than $\gamma_{i}$, which causes cutting interference damaging the manufactured surface. On the contrary, the inclination angle is larger than clearance angle $\gamma_{i+n}$ in right situation, there is no cutting interference for machined surface. Therefore, clearance angle must be larger than inclination angle among all the cutting points.

**Figure 13:**
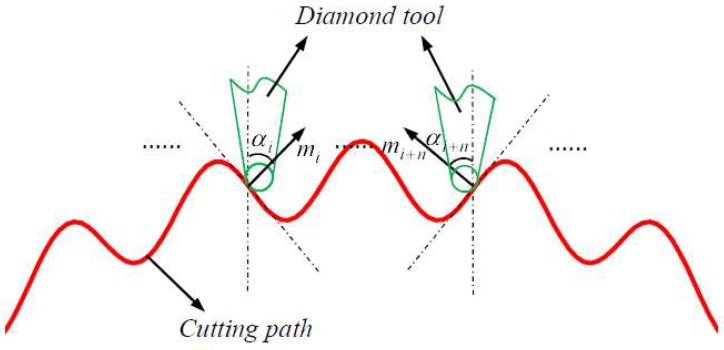
Illustration of wrap angle optimization.

After confirming the advanced double elements, wrap angle is also critical to contribute to surface accuracy, geometry of wrap angle and relation between diamond tool and cutting path are illustrated as Figure 13.

Wrap angle α in Figure 10 shows the geometry details in diamond tool and Figure 13 describes the method to optimize diamond tool wrap angle, on the arbitrary contact points of cutting path, $\vec{m_{i}}$ is the corresponding normal vector, and $\alpha_{i}$ or $\alpha_{i+n}$ is an angle between vertical direction and normal vector direction. According to the geometry depicted in Figure 13, during manufacturing the whole compound freeform surface, diamond tool wrap angle must be larger than double $\alpha_{i\max}$ or equal to it, which can guarantee the machined surface accuracy in superior scale, and it can be described as follows.

(4) $$\alpha \geq 2\alpha_{i\max}$$

where $\alpha_{i\max}=\max\alpha_{i}$.

### 2.4. Tool Nose Radius Compensation

**Figure 14:**
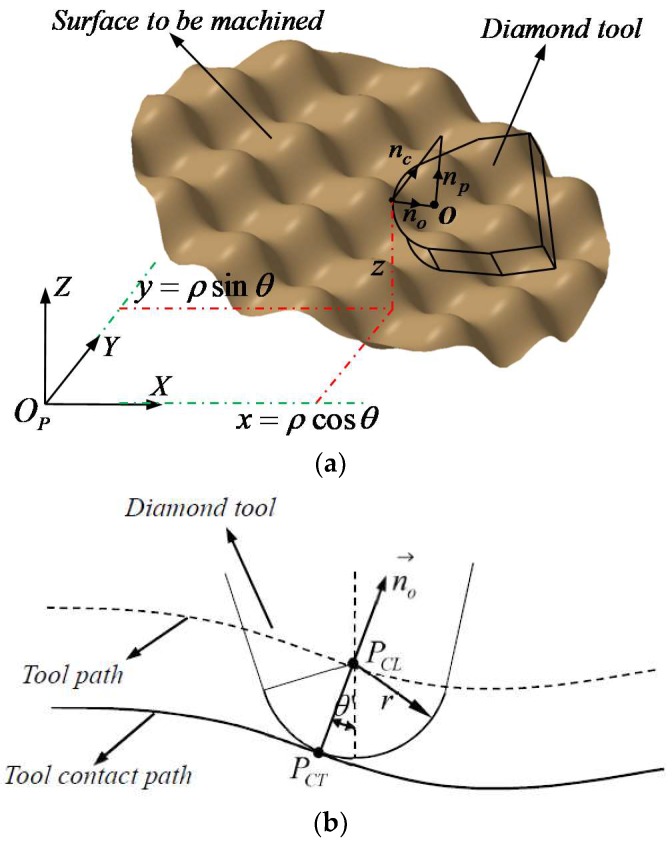
Tool nose radius compensation. (**a**) Tool nose radius compensation model; and (**b**) Tool nose radius compensation presented in detail.

The above sections have investigated tool geometry in detail, this part will explain the tool nose radius compensation. Depending on the actually machining processes, diamond tool nose is not a point in fact, but a circular arc, as for that, it is imperative to make tool nose radius compensation to obtain tool location points, the geometry of compensation is presented as Figure 14. For the sake of calculating tool location points, normal vector of all tool contact points P(x,y,z) is prerequisite for that, which can be expressed as follows:

(5) $$\vec{n_{c}}=(-\partial Z/\partial x,-\partial Z/\partial y,1)=(\cos\alpha^{\prime },\cos\beta^{\prime },\cos\gamma^{\prime })$$

where $\alpha^{\prime }$, $\beta^{\prime }$, $\gamma^{\prime }$ are directional cosine angles. In addition to this, when tool contact point polar angle is θ for the machined points, the cutting plane normal vector $n_{p}$ can be obtained by Equation (6).

(6) $$\vec{n_{p}}=(-\sin\theta ,\cos\theta ,0)$$

The subsequent procedure is to solve the direction of tool nose radius offset, which is expressed as the following equation.

(7) $$\vec{n_{o}}=\vec{n_{c}}-(\vec{n_{c}}\cdot \vec{n_{p}})\vec{n_{p}}$$

The subsequent procedure is to solve the direction of tool nose radius offset, which is expressed as the following equation.

**Figure 15:**
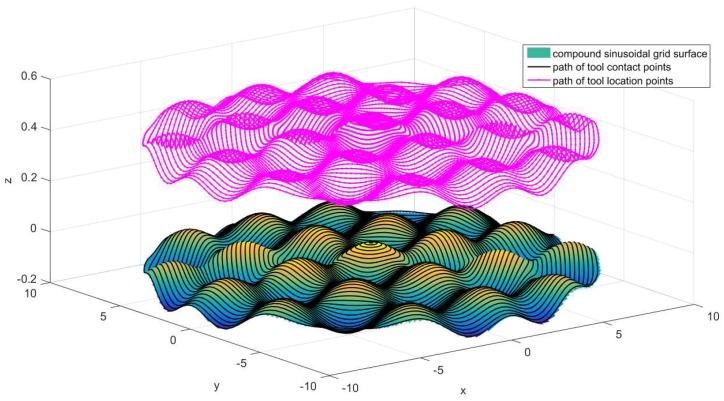
Compensation result of tool nose radius.

All the preparatory works have been accomplished, the last step is tool location points $\left(x^{\prime },y^{\prime },z^{\prime }\right)$ calculation, which can be shown as follows.

(8) $$\left\{ \begin{array}{l} x^{\prime }=x+\frac{\vec{n_{ox}}}{\left\|\vec{n_{o}}\right\|}\cdot r\\ y^{\prime }=y+\frac{\vec{n_{oy}}}{\left\|\vec{n_{o}}\right\|}\cdot r\\ z^{\prime }=z+\frac{\vec{n_{oz}}}{\left\|\vec{n_{o}}\right\|}\cdot r \end{array}\right.$$

where $\vec{n_{ox}},\vec{n_{oy}},\vec{n_{oz}}$ is the component of $\vec{n_{o}}$ in X, Y, Z direction, *r* is tool nose radius. Figure 15 shows the result of tool nose radius compensation.

### 2.5. Error Analysis

The desired surface compound sinusoidal grid surface can be fabricated on the basis of advanced study, however, there is deviation between the ideal surface and the surface manufactured in reality, including two types of surface errors, named cutting residual error and cutting linearization error. The following section is to analyze them.

**Figure 16:**
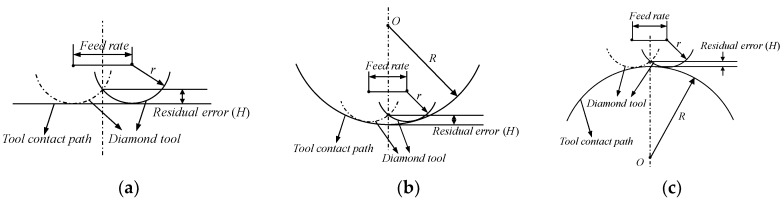
Illustration of cutting residual error. (**a**) Zero curvature surface processing; (**b**) Positive curvature surface machining; (**c**) Negative curvature surface fabricating.

Cutting residual error is analyzed in the first place, which is caused by the cutting path along feed direction, and it is dominant by tool nose radius and feed rate, schematic diagram of cutting residual error is illustrated in Figure 16, including three situations.

Figure 16a is the situation that the curvature of the machined surface point is equal to zero, in this condition, cutting residual error is expressed as follows on the basis of schematic diagram (a).

(9) $$H=r-\sqrt{r^{2}-{\left(f/2\right)}^{2}}$$

where *H* is cutting residual error, *r* is tool nose radius and *f* is feed rate.

Figure 16b is the case that the curvature of manufactured surface point is more than zero, according to the geometry diagram of (b), cutting residual error can be obtained as following.

(10) $$H=R-\sqrt{{\left(R-r\right)}^{2}-{\left(f/2\right)}^{2}}-\sqrt{r^{2}-{\left(f/2\right)}^{2}}$$

where *R* is the radius of curvature at the point between adjacent tool trajectory.

Figure 16c describes the condition that the curvature of fabricated surface point is less than zero, in which cutting residual error is obtained as Equation (11).

(11) $$H=\sqrt{{\left(R+r\right)}^{2}-{\left(f/2\right)}^{2}}-\sqrt{r^{2}-{\left(f/2\right)}^{2}}-R$$

The cutting residual error including three situations has been explained in above section, and we can restrain the cutting residual error by optimize tool nose radius and feed rate parameters to meet the requirement of accuracy based on Equations (9)–(11), while the tool nose radius is confirmed, feed rate is the critical parameter to ensure the desired surface accuracy. Therefore, in the light of Equations (9)–(11) which can be used to do inverse computation to obtain the optimized feed rate, the corresponding equations of (9)–(11) are as follows.

(12) $$\left\{ \begin{array}{l} f_{1}=2\sqrt{-H(H-2r)}\\ f_{2}=\sqrt{-H(H-2R)(H-2r)(H-2R+2r)}/(H-R)\\ f_{3}=\sqrt{-H(H+2R)(H-2r)(H+2R+2r)}/(H+R) \end{array}\right.$$

Based on Equation (12), the required feed rate can be expressed as the following:

(13) $$f\leq \min\{f_{1},f_{2},f_{3}\}$$

**Figure 17:**
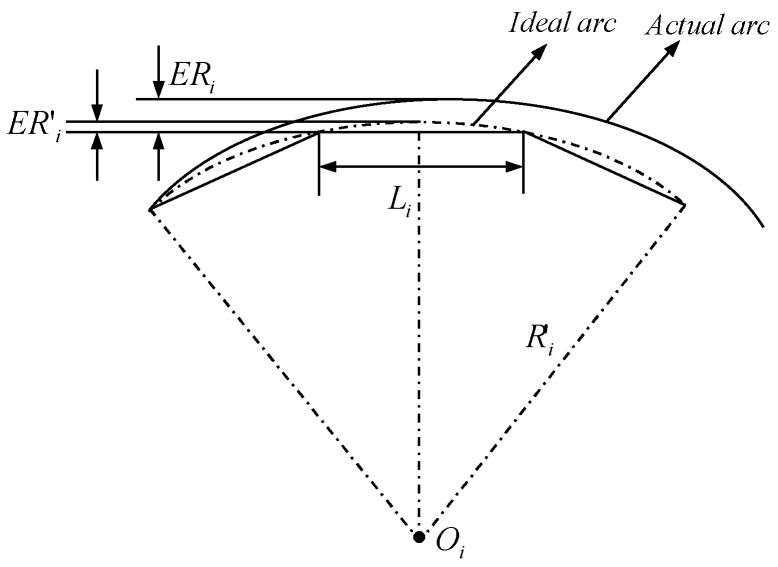
Schematic diagram illustration of cutting linearization error.

In addition to the cutting residual error, the subsequent section is to analyze cutting linearization error that is the other machining error that is generated in the cutting direction in the course of machining, which is due to the distinction between the ideal surface profile and the linear cutting trajectory, it relies mainly upon the distance between adjacent points along the cutting direction. Figure 17 illustrates the schematic diagram of cutting linearization error.

From the schematic diagram illustration of cutting linearization error, which uses the approximate concept that an arc between adjacent points is approximate to an ideal arc, therefore, the cutting linearization error is obtained, as follows, based on the approximate concept and the schematic diagram of cutting linearization error.

(14) $$ER_{i}{}^{\prime }\approx ER_{i}={R^{\prime }}_{i}-\sqrt{{R^{\prime }}_{i}{}^{2}-{\left(L_{i}/2\right)}^{2}}$$

where $ER_{i}$ is cutting linearization error in reality, $E{R^{\prime }}_{i}$ is cutting linearization error according to the approximate concept, ${R^{\prime }}_{i}$ is curvature radius of the ideal arc, $L_{i}$ is the distance between adjacent points. To restrain the cutting linearization error by Equation (14) to optimize the dense of cutting points, which makes the accuracy meet the demand, and the desired $L_{i}$ is shown as the follows.

(15) $$L_{i}=\sqrt{8{R^{\prime }}_{i}\cdot h-4h^{2}}$$

where *h* is the demand cutting linearization error.

## 3. Experiments and Discussion

### 3.1. Experimental Setup

A compound sinusoidal grid surface expressed by Equation (3) with *r*_1_ = *r*_2_ = 1000, $h_{1}=h_{2}=0.03$ was fabricated by Nanoform250 machining system (Precitech, Keene, NH, USA). Geometrical accuracy of the machine is listed in Table 1. Material of workpiece is Al6061, the parameters of applied diamond tool and the restraint of error are listed in Table 2. Machining parameters are presented in Table 3.

Before manufacturing the workpiece, the critical procedure is to center tool by the UltraSet Optical Tool Setter (USOTS) and to balance the spindle that ensures the machining precision of the desired surface. The applied cutting path shown in Figure 8 is employed to fabricate the compound sinusoidal grid surface.

In order to verify the effectivity of the proposed method, the compound freeform surface roughness and form accuracy were measurement and evaluated with a precision form measurement system Taylor Hobson (Leicester, UK) and a high magnification microscope Keyence VR-3200 (Keyence, Osaka, Japan), respectively.

### 3.2. Results and Discussion

**Figure 18:**
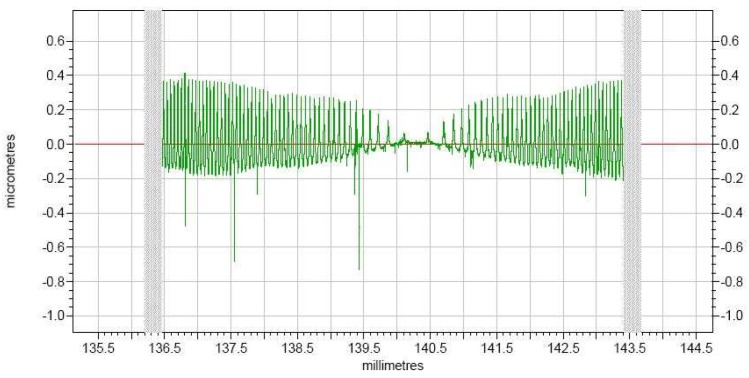
The measured values of compound freeform surface roughness.

The machining activity was conducted using Nanoform250 ultra-precision machine tool. The actual roughness measurement of the compound sinusoidal grid surface is done in a Taylor Hobson. Figure 18 displays the surface roughness, from which we can know that the whole compound freeform surface roughness is well-proportioned, the roughness value achieves 89 nm, and it has a superior surface roughness.

**Figure 19:**
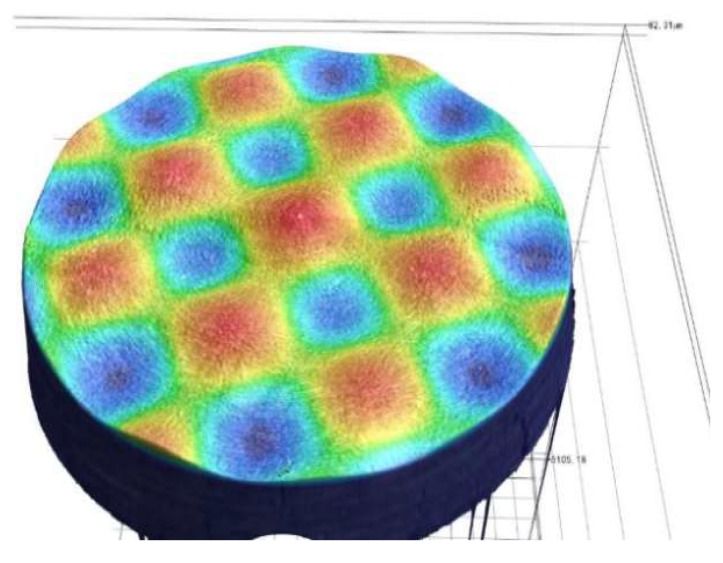
The surface topography of compound sinusoidal surface.

**Figure 20:**
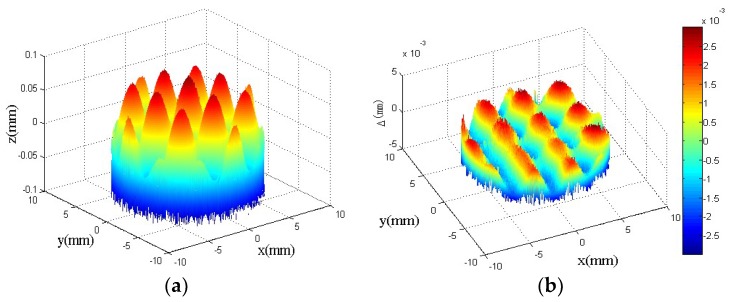
The measured results of PV values. (**a**) The model matching; (**b**) The deviation of matching.

The surface topography of the compound sinusoidal grid surface measured by Keyence VR-3200 is shown in Figure 19, and the surface topography data is obtained at the same time, which is used to be matched with the designed compound sinusoidal grid surface to evaluate the form accuracy. The model matching and deviation are shown in Figure 20a,b, which demonstrate the compound freeform surface form accuracy, it can be observed that the fabricated compound freeform surface PV value is 4.25 μm, and it has a high surface form accuracy.

From the above experiments results, we can know that a compound sinusoidal grid surface is successfully fabricated with well surface finish, which is based on former critical procedures. Above all, the compound freeform surface recombination theory must be acquainted as you expect to machine compound surfaces, because a component with well machined surface needs sufficient theoretical knowledge in order to sustain. Then, an appropriate fabrication method is another vital element, SPDT with STS used in this paper is suitable and highly efficient when compared with the integrated solution (combining fast tool servo with slow tool servo) and laser direct writing method or other solutions, the experiment results have demonstrated that STS is well enough to manufacture compound freeform surfaces. The cutting tool path generation is another indispensable part, most of the research teams acquired failing or defective surfaces on account of inadequate preparation about cutting tool path. A cutting tool path of combining constant angle with constant arc-length path is adopted to process compound sinusoidal grid surface, which contains all the benefits of the two sorts of cutting paths. Finally, a machining error prediction model is necessary to forecast the coming error for designed compound freeform surface, a majority of teams fabricated components by means of trying cutting to guarantee desired surface precision, which is attached with a number of uncertain factors. When compared with the published literature, Xiaodong Zhang et al. studied the mathematic and optical model to propose a grid machining method for the fabrication of compound eye lens. They presented an off-centering machining configuration to avoid the shape distortion that is caused by tool un-alignment. Based on the proposed methods, a compound freeform surface that was constructed by sphere base surface and compound eye lens was successfully machined, but they did not show the surface accuracy and just indicated that the method presented is suitable for the compound eye lens with curve substrate. As far as the author is concerned, there are more and more application fields for compound surfaces with the development of society and technology, and the demand for surface precision will be also greatly improved, so more researches should be done to focus on how to design and machine some compound freeform surfaces.

## 4. Conclusions

In this paper, the following conclusions can be obtained. Firstly, a new recombination method of compound freeform surfaces is presented that demonstrates the method to achieve a compound freeform surface. Secondly, SPDT assisted with STS is presented in order to successfully manufacture the compound sinusoidal grid surface. Thirdly, a cutting tool path that recombines the constant angle cutting path and the constant arc-length cutting path is proposed to manufacture the compound freeform surface, which can ensure the surface precision. Finally, a fabrication error prediction model is established for forecasting the machining deviation in order to obtain the optimum cutting path parameters, which avoids trying cutting to achieve an ideal surface finish when compared with the other academic research. By cutting an Al6061 workpiece and measuring the machined surface, it is found that the roughness value and PV value of the machined compound freeform surface can be 89 nm and 4.25 μm, respectively. Obviously, the theoretical analysis and experimental results demonstrate that the proposed methods are feasible to machine a compound freeform surface and can improve the machining precision.

## Figures and Tables

**Table 1:** Performance parameters of machine tool.

Parameters	Values
Travel of *X*- and *Z*-axis	220 mm
Straightness of *X*- and *Z*-axis	0.2 μm
Feedback resolution of *X*- and *Z*-axis	0.016 nm
Feedback resolution of *C*-axis	0.026 arcsec
Position accuracy of *C*-axis	+/−2 arcsec

**Table 2:** Diamond tool parameters and error restraint value.

Parameters	Values
Rake angle	0°
Clearance angle	10°
Wrap angle	120°
Tool nose radius	0.506 mm
Cutting residual error bound	0.01 mm
Cutting linearization error bound	0.01 mm

**Table 3:** Machining parameters in manufacturing the compound surface.

Name of Parameters	Value
Depth of cut	5 μm
Spacing of revs	0.05 mm
Cutting time	16 min
Number of the cutting points	80,000
Acceleration of slide axes	0.2 g
Interpolated method	PVT

## Nomenclature

STS	Slow tool servo	
SPDT	Single point diamond turning	
PV	Peak valley value	
FTS	Fast tool servo	
*Z*_1_	Base surface	Equation (1)
*r*_1_	Confirm the curvature in x direction	Equation (1)
*r*_2_	Confirm the curvature in y direction	Equation (1)
*Z*_2_	Feature surface	Equation (2)
*F*_1_	Define the sinusoidal grid surface along X direction	Equation (2)
*F*_2_	Define the sinusoidal grid surface along Y direction	Equation (2)
*h*_1_	Confirm the amplitude of the sinusoidal grid surface along X direction	
*h*_2_	Confirm the amplitude of the sinusoidal grid surface along Y direction	
*ω*_1_	Confirm the period of the sinusoidal grid surface along X direction	
*ω*_2_	Confirm the period of the sinusoidal grid surface along Y direction	
*Z*	Define the compound sinusoidal grid surface	Equation (3)
Δ*θ*	Constant angle of constant angle cutting path	Figure 6
Δ*S*	Constant arc-length of constant arc-length cutting path	Figure 7
*r*	Diamond tool nose radius	Figure 10
*γ*	Diamond tool clearance angle	Figure 10
*α*	Diamond tool wrap angle	Figure 10
*r_i_*	Diamond tool nose radius	Figure 11
*ρ*	Polar radius	Figure 11
*γ_i_*	Diamond tool nose clearance angle	Figure 12
*Q_i_*	Cutting points	Figure 12
*α_i_*	Diamond tool nose wrap angle	Figure 13
*m_i_*	Normal vector on arbitrary cutting points	Figure 13
$\alpha_{i\max}$	$=\max\alpha_{i}$	
$\vec{n_{c}}$	Normal vector of tool contact points	
*α*′	Directional cosine angle between *n*_c_ and X direction	
*β*′	Directional cosine angle between *n*_c_ and Y direction	
*γ*′	Directional cosine angle between *n*_c_ and Z direction	
*θ*	Tool contact points polar angle	
$\vec{n_{p}}$	Cutting plane normal vector	
$\vec{n_{o}}$	Tool nose radius offset direction	
(*x*′, *y*′, *z*′)	Tool location points	Equation (8)
$\vec{n_{ox}}$	Component of $\vec{n_{o}}$ along X direction	Equation (8)
$\vec{n_{oy}}$	Component of $\vec{n_{o}}$ along Y direction	Equation (8)
$\vec{n_{oz}}$	Component of $\vec{n_{o}}$ along Z direction	Equation (8)
*P_CL_*	Tool location points	Figure 14b
*P_CT_*	Tool contact points	Figure 14b
*θ*′	Inclination angle of the contact points	Figure 14b
*H*	Residual error	Figure 16
*R*	Curvature radius	Figure 16
*f*	Feed rate	Equation (9)
*f_1_*, *f_2_*, *f_3_*	Inverse computation results based on Equations (9)–(11)	Equation (12)
*R*′*_i_*	Curvature radius of ideal arc	Figure 17
*L_i_*	Distance between adjacent points	Figure 17
*ER* *′*	Cutting linearization error	Figure 17
*ER*	Cutting linearization error	Figure 17
*h*	Demand cutting linearization error	Equation (15)
PVT	Position velocity time interpolation	
USOTS	UltraSet optical tool setter	

## References

[1] Zhu W.L., Duan F., Zhang X., Zhu Z.W., Ju B.F. (2018). A new diamond machining approach for extendable fabrication of micro-freeform lens array. Int. J. Mach. Tools Manuf..

[2] Wang Y., Lu Z., Liu H. (2007). Application of freeform surface prism. Infrared Laser Eng..

[3] Fang F.Z., Zhang X.D., Weckenmann A., Zhang G.X., Evans C. (2013). Manufacturing and measurement of freeform optics. CIRP Ann.-Manuf. Technol..

[4] Jiang X., Scott P., Whitehouse D. (2007). Freeform surface characterisation-a fresh strategy. CIRP Ann.-Manuf. Technol..

[5] Ren M.J., Cheung C.F., Kong L.B. (2011). A robust surface fitting and reconstruction algorithm for for characterization of ultra-precision freeform surfaces. Measurement.

[6] Cheng Y., Fang F., Zhang X. (2012). Design and manufacture of off-axis optical reflective integrator with faceted structure. Opt. Eng..

[7] Zhang X.D., Fang F.Z., Wu Q.Q., Liu X.L., Gao H.M. (2013). Coordinate transformation machining of off-axis aspheric mirrors. Int. J. Adv. Manuf. Technol..

[8] Ji S., Yu H., Zhao J., Liu X., Hu M. (2016). Analysis and comparison of two different ultra-precision manufacturing methods for off-axis parabolic mirror with single point diamond turning. Proc. Inst. Mech. Eng. Part B J. Eng. Manuf..

[9] Zhang X.D., Fang F.Z., Wang H.B., Wei G.S., Hu X.T. (2009). Ultra-precision machining of sinusoidal surfaces using the cylindrical coordinate method. J. Micromech. Microeng..

[10] Hong L.U., Deug-Woo L.E.E., Sang-Min L.E.E., Jeong-Woo P.A.R.K. (2012). Diamond machining of sinusoidal grid surface using fast tool servo system for fabrication of hydrophobic surface. Trans. Nonferr. Met. Soc. China.

[11] Zhou X., Zuo C., Liu Q., Wang R., Liu J. (2016). Development of a double-frequency elliptical vibration cutting apparatus for freeform surface diamond machining. Int. J. Adv. Manuf. Technol..

[12] Kong L.B., Cheung C.F. (2012). Modeling and characterization of surface generation in fast tool servo machining of microlens arrays. Comput. Ind. Eng..

[13] Zhang Q., Zhao Q., To S., Guo B., Rao Z. (2018). Precision machining of ‘water-drop’ surface by single point diamond grinding. Precis. Eng..

[14] Ji S., Yu H., Zhao J., Liu X., Zhao M. (2016). Ultra-Precision Machining of a Large Amplitude Sinusoidal Ring Surface Based on a Slow Tool Servo. Stroj. Vestnik J. Mech. Eng..

[15] Wojciechowski S., Maruda R.W., Krolczyk G.M., Niesłony P. (2018). Application of signal to noise ratio and grey relational analysis to minimize forces and vibrations during precise ball end milling. Precis. Eng..

[16] Kumar J., Negi V.S., Chattopadhyay K.D., Sarepaka R.V., Sinha R.K. (2017). Thermal effects in single point diamond turning: Analysis, modeling and experimental study. Measurement.

[17] Wojciechowski S., Maruda R.W., Barrans S., Nieslony P., Krolczyk G.M. (2017). Optimisation of machining parameters during ball end milling of hardened steel with various surface inclinations. Measurement.

[18] Zhang X., Fang F., Yu L.H., Jiang L., Guo Y. (2013). Slow slide servo turning of compound eye lens. Opt. Eng..

